# Special Issue “Pulmonary and Critical Care Practice in the Pandemic of COVID-19”

**DOI:** 10.3390/jcm11051336

**Published:** 2022-02-28

**Authors:** Jihad Mallat

**Affiliations:** 1 Critical Care Institute, Cleveland Clinic Abu Dhabi, Abu Dhabi 112412, United Arab Emirates; mallatjihad@gmail.com; 2 Cleveland Clinic Lerner College of Medicine of Case, Western Reserve University, Cleveland, OH 44195, USA; 3 Faculty of Medicine, Normandy University, UNICAEN, ED 497, 1400 Caen, France

Severe acute respiratory syndrome coronavirus-2 (SAR-CoV-2), which is responsible for the coronavirus disease 2019 (COVID-19), has hit the world as a global pandemic at an unparalleled scale [1], causing substantial morbidity and mortality and inflicting unprecedented harm on the economic and health sectors [2]. Therefore, intensive efforts have been made worldwide to develop effective therapies to reduce the risk of severe COVID-19 illness, hospitalizations, and deaths. To address this topic, this Special Issue in the Journal of Clinical Medicine (JCM) is dedicated to the collection of high-quality scientific papers, primarily focused on critical care practices during the pandemic to enhance our understanding and provide information on treatment approaches to improve the management and outcomes of these critically ill COVID-19 patients.

In this Special Issue, Thakur et al. [3] showed that SARS-CoV-2 could theoretically infect various organs after binding to the ubiquitous angiotensin-converting enzyme-2 cell membrane responsible for myocardial dysfunction, gastrointestinal symptoms, hepatic and renal injuries, dermatological complications, and neurological illnesses. However, due to the airborne nature of the infectious agent, the respiratory system is still the most commonly affected. The clinical picture is very heterogeneous, but the potential for severe life-threatening conditions in adults stems from lung damage since inflammatory processes causing airway, alveolar and vascular dysfunction and injury can lead to rapidly progressive acute respiratory distress syndrome (ARDS). Corticosteroids might mitigate this exacerbated inflammatory response by inhibiting the expression of proinflammatory cytokines [4]. The RECOVERY trial demonstrated that the use of dexamethasone reduced mortality, especially in the subgroup of severe COVID-19 patients requiring high oxygen therapy and invasive mechanical ventilation [5]. Methylprednisolone has better lung penetration, lower potent anti-inflammatory effects, and shorter plasma half-time than dexamethasone. In this Special Issue, Badr et al. found that methylprednisolone treatment in severe COVID-19 ARDS mechanically ventilated patients was independently associated with a longer number of days alive and free from mechanical ventilation during the first 28 days [6].

Convalescent plasma (CP) from recovered patients is believed to provide passive immunity against viral infections, and it has resurfaced again as a potential treatment of many viral illnesses, including SARS, MERS, with inconclusive results [7,8]. However, in severe COVID-19 patients, CP treatment was not associated with the time to clinical improvement or death [9]. These findings were confirmed in different randomized trials, including the RECOVERY and REMAP-CAP trials [10,11]. Other aspects of COVID-19 ARDS management were also addressed in this Special Issue [12,13]. In ARDS, the prone position (PP) is commonly used to increase oxygenation, with the overall goal of minimizing ventilator-induced lung damage. The PP allows a better distribution of the transpulmonary pressure, relieves the lungs behind the heart, and improves lymphatic drainage. Interestingly, Parker et al. [12] showed that a single PP duration of >39 h was safe and effective, sparing the burden of multiple prone position sessions. Furthermore, there was no significant advantage in initiating PP when the PaO_2_/FiO_2_ ratio was >150 mmHg [12]. The effects of tracheostomy techniques and timing on the outcomes of COVID-19 patients were also investigated. In this multicenter retrospective study, Battaglinin et al. showed that among critically ill COVID-19 patients, neither early (less than 15 days) nor percutaneous tracheostomy improved outcomes, but they did shorten intensive care unit length stay. Infectious complications were less frequent with percutaneous than surgical tracheostomy [14].

In this Special Issue, Ghosn et al. found that severe acute kidney injury (AKI) was common in COVID-19 critically ill patients and was not linked to inflammatory or thrombotic markers. Additionally, in these patients, severe AKI was independently associated with hospital mortality and hospital length of stay [15]. Usually, these patients need renal replacement therapy for a long time, requiring a switch from a non-tunneled dialysis catheter to a tunneled dialysis catheter (TDC), which is commonly executed under fluoroscopic guidance to lower catheter-related complications. However, this necessitates moving patients outside the intensive care unit, potentially exposing many healthcare providers to COVID-19. Interestingly, Sohail et al. defined a bedside right internal jugular TDC insertion approach in COVID-19 patients, employing ultrasound and anatomic landmarks without fluoroscopic guidance, possibly diminishing the risk of COVID-19 transmission among healthcare workers without jeopardizing patient security or catheter function [16].

Several other interesting findings were also published in this Special Issue [17,18,19]. As the Guest Editor, I would like to give special thanks to the reviewers for their professional comments and to the JCM team for their robust support. Finally, I sincerely thank all the authors for their valuable contributions.

## Abbreviations

SAR-CoV-2	severe acute respiratory syndrome coronavirus-2
COVID-19	coronavirus disease 2019
ARDS	acute respiratory distress syndrome
AKI	acute kidney injury
PP	prone position
TDC	tunneled dialysis catheter
CP	convalescent plasma
JCM	Journal of Clinical Medicine
PaO_2_	arterial oxygen pressure
FiO_2_	inspiratory oxygen fraction
